# Fabrication and application of an ionic liquid–agarose–glycerin composite for the liquid phase microextraction of fluoroquinolones from water and wastewater samples prior to HPLC-PDA

**DOI:** 10.1039/d6ra06858h

**Published:** 2026-10-23

**Authors:** Amal Al-Ghrairi, Saeed Mohammad Sorouraddin, Mohammad Reza Afshar Mogaddam, Mir Ali Farajzadeh

**Affiliations:** a Department of Analytical Chemistry, Faculty of Chemistry, University of Tabriz Tabriz Iran Saied_sorour@yahoo.com +98 41 33393110; b Pharmaceutical Chemistry Branch, College of Pharmacy, University of Misan Iraq; c Food and Drug Safety Research Center, Pharmaceutical Sciences Institute, Tabriz University of Medical Sciences Tabriz Iran mr.afsharmogaddam@yahoo.com; d New Material and Green Chemistry Research Center, Khazar University 41 Mehseti Street Baku AZ1096 Azerbaijan; e Pharmaceutical Analysis Research Center, Pharmaceutical Sciences Institute, Tabriz University of Medical Sciences Tabriz Iran; f Engineering Faculty, Near East University Mersin 10 Nicosia 99138 North Cyprus Turkey

## Abstract

In the present study, a new extraction procedure was developed by preparing a cylinder gel composed of agarose, glycerol, and a hydrophobic ionic liquid. The agarose–glycerol matrix serves as a solid support that entraps the ionic liquid within its three-dimensional network; upon heating, the gel gradually softened and released the ionic liquid into the aqueous solution of analytes, where it acted as a micro-extractant. This approach was applied for the simultaneous preconcentration and extraction of two fluoroquinolone antibiotics, ciprofloxacin and enrofloxacin, from water and wastewater samples. Key parameters affecting extraction efficiency, including ionic liquid type, ionic liquid loading volume, salt concentration, sample temperature, pH, number of gel migration cycles, and elution solvent type and volume, were systematically optimized. Under the optimal conditions, limits of detection for ciprofloxacin and enrofloxacin were 0.09 and 0.06 ng mL^−1^ and limits of quantification were 0.29 and 0.19 ng mL^−1^, respectively. The linear ranges of calibration curves were 0.29–150 and 0.19–150 ng mL^−1^ with determination coefficients of 0.991 and 0.985, respectively. The extraction recoveries were 79 ± 4% for ciprofloxacin and 87 ± 5% (*n* = 3) for enrofloxacin. The method was validated in real samples (pharmaceutical wastewater, tap water, river water, and dam water) spiked at two levels (5 and 25 ng mL^−1^ of each analyte), yielding relative recoveries in the range of 89–107%, confirming its effectiveness in complex matrices. The proposed technique offered high sensitivity, acceptable repeatability, and negligible organic solvent consumption, making it a green and efficient alternative for monitoring antibiotic residues in environmental waters.

## Abstract 2

A new extraction procedure was developed by preparing a cylinder gel composed of agarose, glycerol, and a hydrophobic ionic liquid.

## Graphical abstract

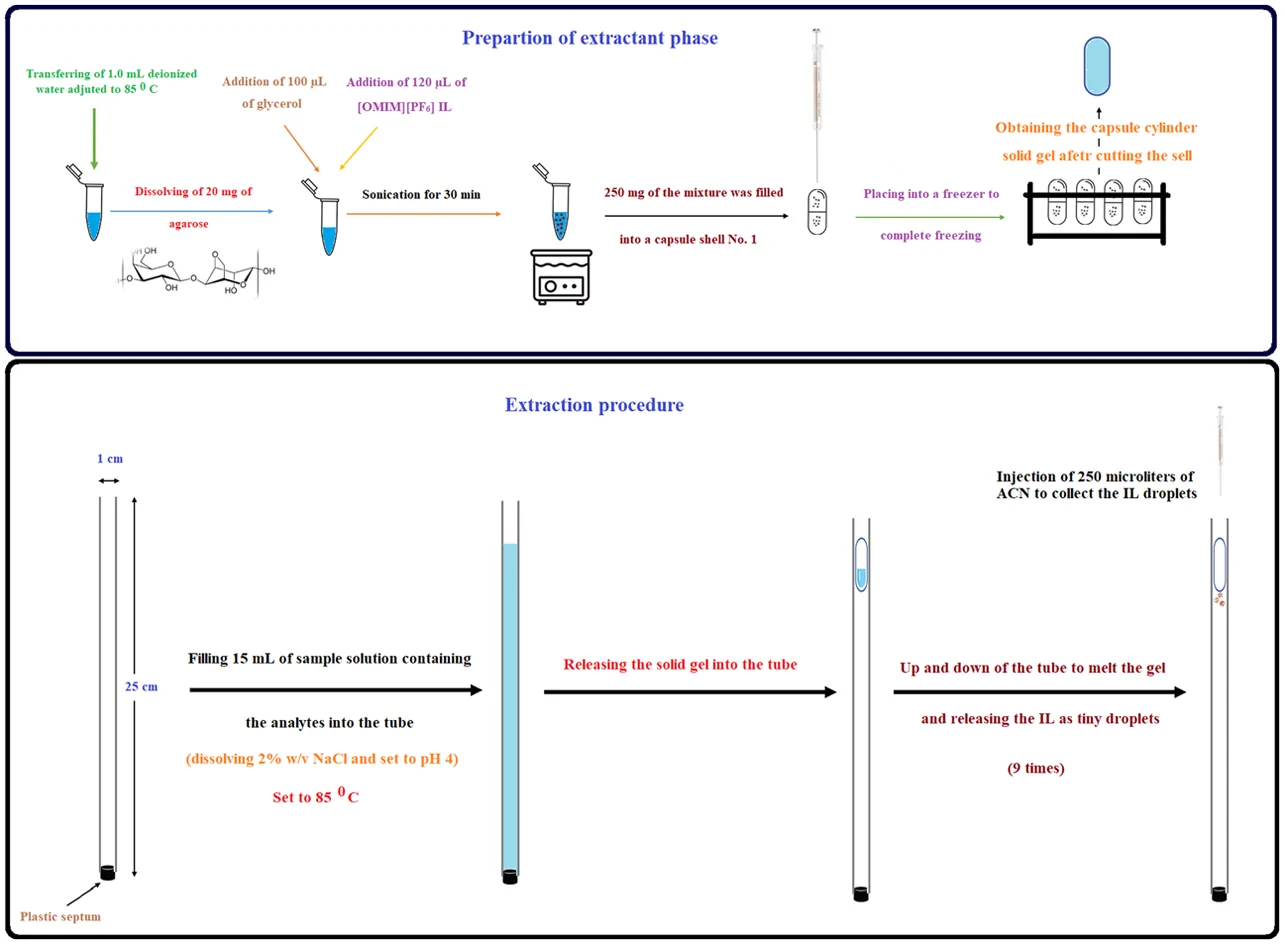

## 1. Introduction

The occurrence of pharmaceutical residues in aquatic environments has become a major concern for public health and ecological sustainability. Among these contaminants, antibiotics are particularly problematic due to their widespread consumption in human and veterinary medicine, incomplete metabolism, and continuous discharge into surface and groundwaters.^1^ Fluoroquinolones, such as ciprofloxacin and enrofloxacin, are among the most frequently prescribed antibiotics owing to their broad-spectrum antibacterial activity.^2^ Even at trace levels, their persistence in the environment can promote antimicrobial resistance and exert toxic effects on aquatic organisms.^3^ Therefore, reliable and routine monitoring of these pollutants in diverse environmental matrices is essential for effective risk assessment and quality control. The development of analytical methods that combine sufficient accuracy and sensitivity with applicability to complex matrices remains a central challenge in analytical chemistry. Sample preparation step is particularly critical because it directly affects accuracy, precision, detection limits, and reproducibility.^4–6^ Notably, the overall performance of any instrumental system-whether high performance liquid chromatography (HPLC) or mass spectrometry-is highly dependent on the effectiveness of sample preparation, as even the most sophisticated detectors cannot provide trustworthy results from untreated or contaminated samples. Although classical techniques such as liquid–liquid extraction (LLE) and solid phase extraction (SPE) have been widely used, they are associated with significant drawbacks including high solvent consumption, lengthy procedure, large waste generation, high cost, and poor automation compatibility.^7–9^ These limitations have motivated the development of greener, faster, and more cost-effective extraction strategies. In recent years, various microextraction techniques, including liquid phase microextraction, solid-phase microextraction, and microscale solid phase extraction (µ-SPE), have been introduced to address these challenges.^10–12^ However, some of these methods still suffer from drawbacks such as expensive instrumentation, complex operation, or limited phase stability. Consequently, the design of new extraction phases that are inexpensive, biocompatible, and highly efficient remains an active area of research within green analytical chemistry.

Natural polymers such as agarose have attracted considerable interest due to their biodegradability, availability, low cost, and ease of modification.^13,14^ Agarose is a linear polysaccharide composed of alternating d-galactose and 3,6-anhydro-l-galactopyranose units. It dissolves in hot water (approximately at 85 °C) and, upon cooling below its gelation temperature (35–40 °C) forms a stable three-dimensional porous network through hydrogen bonding and hydrophobic interactions. The resulting hydrogel exhibits tunable pore sizes, excellent mechanical stability, and high biocompatibility, making it an ideal scaffold for the physical entrapment of various extraction media, including ionic liquids (ILs) and deep eutectic solvents.^15^ ILs have been emerged as a new class of environmentally friendly solvents, characterized by negligible vapour pressure, high thermal stability, and tunable physicochemical properties.^16,17^ Hydrophobic ILs containing hexafluorophosphate anion ([PF_6_]^−^) have shown remarkable efficiency in extracting pharmaceuticals and organic pollutants through hydrophobic, hydrogen-bonding, and electrostatic interactions.^18^ Glycerol, when added to the agarose matrix, functions as a plasticizer and viscosity modifier, improving the mechanical resilience and permeability of the gel while reducing its brittleness.^19^

In the present approach, an agarose–glycerol matrix serves not as a sorbent but as a controlled-release carrier for an IL. When the gel is placed into a heated aqueous solution of the analytes, the agarose network gradually softens and releases the entrapped IL into the solution. The released IL then acts as a micro-extractant, partitioning the target analytes from the aqueous phase into the IL through LLE. This mechanism is fundamentally different from conventional SPE, where analytes are adsorbed onto a solid surface. The released IL, extracts the analytes and then it is collected by addition of acetonitrile, and the resulting organic phase is directly injected into HPLC-photodiode array (PDA) system. The aim of the present study is to develop and evaluate a new extraction method based on the agarose/glycerol/IL composite gel that functions as a controlled-release extraction medium for the simultaneous extraction of ciprofloxacin and enrofloxacin from water and wastewater samples. The method offers operational simplicity, rapidity, low cost, minimal organic solvent consumption, and easy coupling with chromatographic method.

## 2. Experimental

### 2.1. Chemicals and solutions

Standards of ciprofloxacin, enrofloxacin, and levofloxacin (internal standard, IS) were obtained from Merck (Darmstadt, Germany). Analytical-grade agarose powder, glycerol, ILs including 1-butyl-3-methylimidazolium hexafluorophosphate ([BMIM][PF_6_]), 1-hexyl-3-methylimidazolium hexafluorophosphate ([HMIM][PF_6_]), 1-octyl-3-methylimidazolium hexafluorophosphate ([OMIM][PF_6_]), and HCl (37% w/w), NaCl, triethanolamine, acetonitrile, and NaOH were all purchased from Merck. A stock solution of the target antibiotics (ciprofloxacin and enrofloxacin) was prepared in methanol at a concentration of 1000 mg L^−1^ (of each). From this stock solution, a series of working standard solutions was subsequently prepared through stepwise dilution with deionized water.

### 2.2. Apparatus

In this study, quantitative analysis of the fluoroquinolone antibiotics was carried out on a HPLC system (Waters Alliance 1525) equipped with a PDA (model 996), allowing for multi-wavelength detection. The separation process was conducted on a reversed-phase C_18_ column (250 mm × 4.6 mm i.d., 3 µm particle size), with the column compartment maintained at a fixed temperature of 30 °C throughout the analysis. The mobile phase, delivered in an isocratic mode at a constant flow rate of 1.0 mL min^−1^, consisted of 85 : 15 (v/v) mixture of acetonitrile and water (pH adjusted to 3.0 with phosphoric acid, 0.1 M). The detector was set at 278 nm for ciprofloxacin and 277 nm for enrofloxacin. A manual injection loop with a fixed volume of 20 µL was employed for introducing the samples into the chromatographic system.

### 2.3. Real samples

To evaluate the proposed method's performance in real samples and assess matrix effect, four water samples with different origins were collected from East Azerbaijan Province, Iran: pharmaceutical wastewater, urban tap water, river water, and dam water. The samples were collected in amber glass bottles, sealed with parafilm, and transported at 4 °C. Upon arrival to laboratory, each sample was filtered through Whatman Grade 42 paper (2.5 µm) to remove suspended particles. Filtered samples were immediately extracted under optimal conditions or stored at 4 °C. All extraction and analytical steps were completed within 24 hours to prevent analytes degradation.

### 2.4. Preparation of composite gel

To prepare composite gel, 20 mg of agarose was dissolved in 1 mL of hot water (85 °C). Then, 120 µL of [OMIM][PF_6_] and 100 µL of glycerol were added to the solution, and the resulting suspension was placed in an ultrasonic bath for 30 min to obtain a completely homogeneous mixture. Subsequently, 250 mg of the mixture was filled into a capsule shell no. 1 (with a capacity of 250 mg). The capsule was stored in a freezer, and after complete freezing, the shell was removed by a cutter, and the extractant phase (solid gel) was used as the adsorbent for the extraction step.

### 2.5. Extraction procedure

First, 15 mL of deionized water spiked with the target analytes at a concentration of 50 ng mL^−1^ and IS at a concentration of 100 ng mL^−1^, containing NaCl at a concentration of 2% (w/v), adjusted to pH 4, and set at 85 °C was transferred to a narrow tube (i.d. of 1 cm and length of 25 cm). Then, the cylindrical extractant (containing the gel and IL) was released into the tube and moved within the tube (by manually moving up and down of the tube) for 9 times to perform the extraction process during 3 min. After performing the extraction step, 250 µL of acetonitrile was added to the tube to collect the droplets of IL (containing the extracted analytes) dispersed in the solution in the bottom of the tube. The upper organic phase was separated and, after dilution with the mobile phase used in the separation system to a final volume of 100 µL, was injected into the HPLC-PDA system.

### 2.6. Calculations

Extraction recovery (ER) of the antibiotics was calculated using eqn (1):1
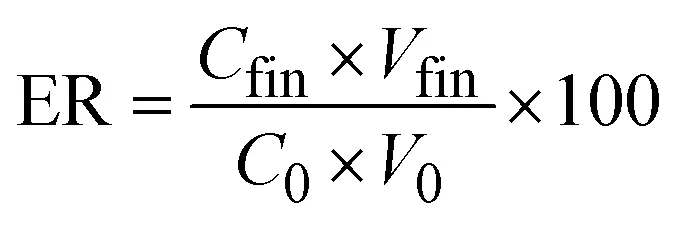
In this equation, *C*_0_ denotes the original concentration of the analyte present in the initial aqueous phase, whereas *C*_fin_ corresponds to the equilibrium concentration of the analyte in the final phase. The volumes of aqueous and final phases are represented by *V*_0_ and *V*_fin_, respectively. The equilibrium concentration of the extracted analyte (*C*_fin_) was obtained from calibration curve.

## 3. Results and discussion

### 3.1. Optimization of parameters affecting extraction efficiency

#### 3.1.1. Selection of IL type

The selection of IL type as the extractive phase plays a decisive role in the method efficiency. In this study, three hydrophobic ILs with different cations, including [BMIM][PF_6_], [HMIM][PF_6_], and [OMIM][PF_6_] were selected and their extraction performance for the target analytes was compared. The results (Fig. 1a) clearly showed that [OMIM][PF_6_] provided the highest chromatographic responses for both ciprofloxacin and enrofloxacin, compared to [BMIM][PF_6_] and [HMIM][PF_6_]. This superiority can be attributed to the longer alkyl chain of the octyl cation, which, enhancing hydrophobic interactions with the quinoline core of the analytes.^19,20^ Additionally, the increased alkyl chain length improves the stability of the extraction phase within the agarose gel matrix. The π–π interactions between the imidazolium ring and the quinoline ring of the analytes are also more efficient in [OMIM][PF_6_] due to more suitable spatial arrangement and higher polarizability. Therefore, [OMIM][PF_6_] was selected as the optimal IL for the further researches.^20–23^

**Fig. 1:**
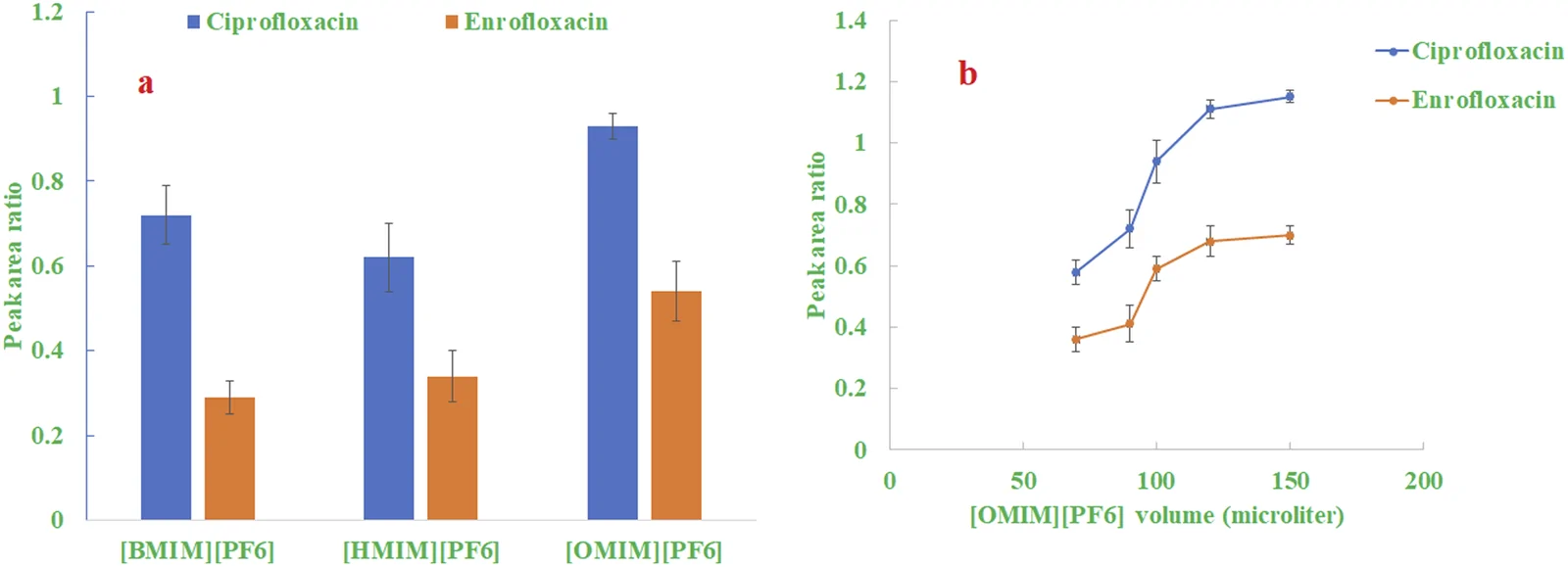
(a) Selection of IL type. Extraction conditions: analytes concentration: 50 ng mL^−1^ of each; IL volume: 100 µL; analytes solution temperature: 70 °C; pH 6; number of extractions: 2; and acetonitrile volume: 200 µL. The analytical response was defined as the ratio of the peak area of each analyte to the peak area of the IS. The standard deviation of three replicate analyses was used for error bar calculations. (b) Optimization of IL volume. Extraction conditions: IL type: [OMIM][PF_6_], and other conditions were described in (a). The analytical response was defined as the ratio of the peak area of each analyte to the peak area of the IS.

#### 3.1.2. Optimization of IL volume

The amount of IL loaded into the gel directly affects the extraction capacity. The effect of IL volume (70–150 µL) on the peak areas of both analytes was investigated (Fig. 1b). Increasing the IL volume from 70 to 150 µL significantly enhances the responses regarding the increasing of extractant to sample solution phase ratio and improving the method efficiency. Although the highest response and observed at 150 µL, the increase in response from 120 to 150 µL for ciprofloxacin and for enrofloxacin is negligible. On the other hand, using a higher IL volume increases the cost of analysis and gel viscosity, which can impair permeability and mass transfer. Therefore, considering economic and performance considerations, 120 µL was selected as the optimal value.

#### 3.1.3. Optimization of salt (NaCl) concentration

Increasing ionic strength through salt addition, typically *via* the salting-out effect, can increase the transfer of analytes into the extractive phase due to reducing of the analyte solubility. In this study, the effect of NaCl concentration was investigated in the range of 0 to 10% (w/v) on extraction efficiency. The results (Fig. 2a) indicated that increasing the salt concentration from 0 to 2% (w/v) significantly increases the analytical signals for both analytes. This increase can be attributed to the salting-out effect owning to reduction of analytes solubility into the aqueous phase, leading to greater transfer to the IL. Increasing the salt concentration to 5% (w/v) do not cause a significant change in the analytical signals. However, at the concentration of 10% (w/v), the responses for both analytes decrease, which may be attributed to the increased solution viscosity, decreased diffusion coefficient of the analytes, and enhanced competitive interactions of salt ions with the analytes. Based on the above observations, NaCl concentration of 2% (w/v) was selected as the optimal condition.

**Fig. 2:**
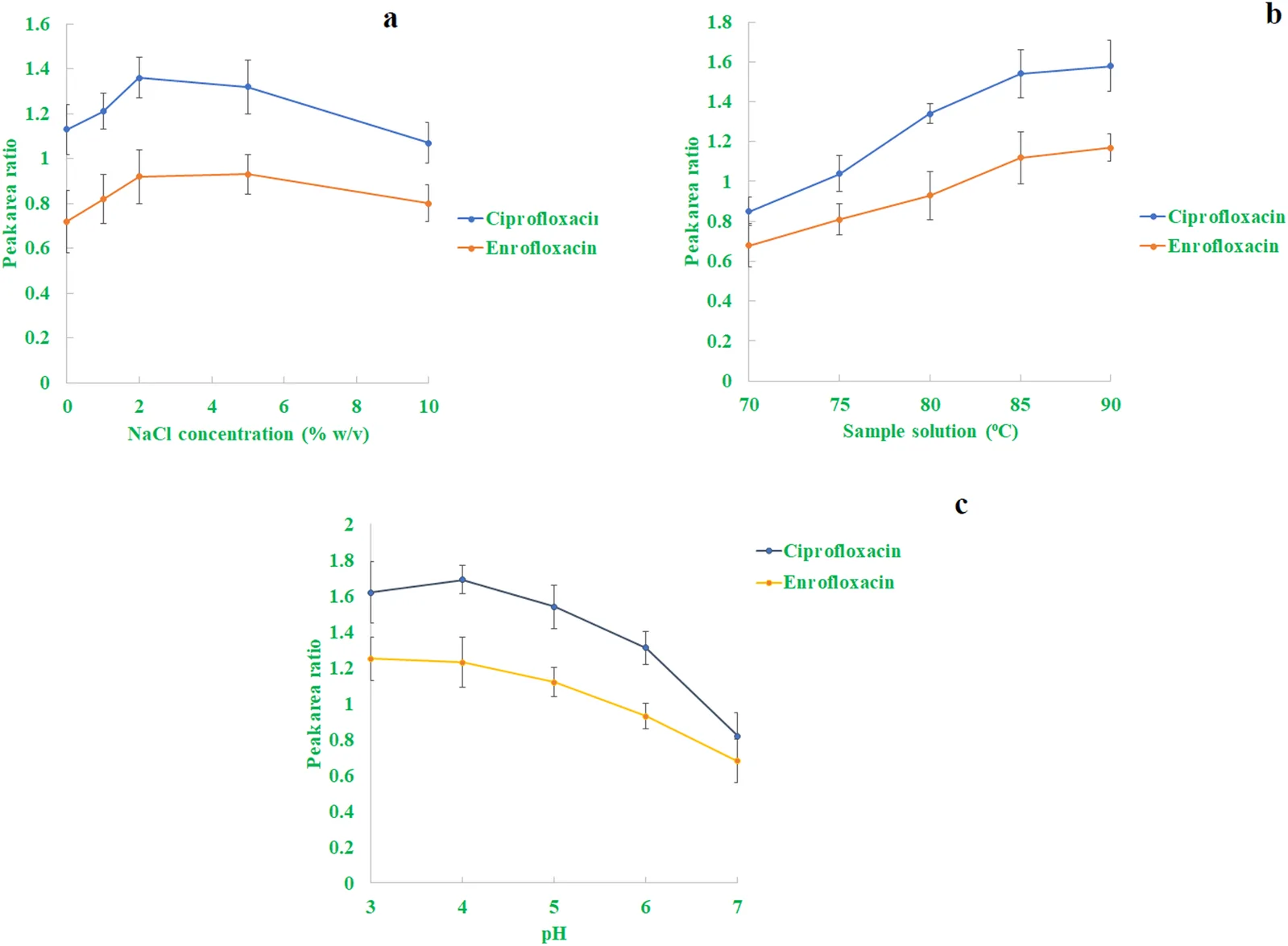
(a) Optimization of NaCl concentration. Extraction conditions: IL volume: 120 µL, and other conditions were described in Fig. 1b. The analytical response was defined as the ratio of the peak area of each analyte to the peak area of the IS. The standard deviation of three replicate analyses was used for error bar calculations. (b) Optimization of sample solution temperature. Extraction conditions: NaCl concentration: 2% (w/v), and other conditions were described in (a). The analytical response was defined as the ratio of the peak area of each analyte to the peak area of the IS. The standard deviation of three replicate analyses was used for error bar calculations. (c) Investigation of pH effect. Extraction conditions: Sample solution temperature: 85 °C, and other conditions were described in (b). The analytical response was defined as the ratio of the peak area of each analyte to the peak area of the IS.

#### 3.1.4. Optimization of sample solution temperature

Temperature plays a dual role: it facilitates the release of IL from the agarose matrix and enhances mass transfer of the analytes. The effect of temperature in the range of 70–90 °C was evaluated (Fig. 2b). Increasing the temperature from 70 to 90 °C significantly improves the peak areas for both analytes. At higher temperatures, the agarose network softens, allowing a more release of the IL. Also, high temperatures reduce viscosity and accelerating diffusion. Temperatures above 90 °C were not tested as they caused decomposition of the gel components. Hence, 85 °C was chosen as the optimal temperature.

#### 3.1.5. Optimization of pH

pH of analytes solution affects the ionization state of analytes, thereby influencing their partitioning into IL. The effect of pH in the range of 3–7 was investigated (Fig. 2c). The responses were approximately constant at pH 3 and 4 for both analytes. At higher pHs (5–7), the responses decrease markedly. This behavior is consistent with the p*K*_a_ values of fluoroquinolones (carboxyl group: ∼6; amino group: ∼8–9). At low pHs, the analytes are predominantly cationic and can form strong ionic and hydrogen-bonding interactions with the IL. At pH 4, both analytes exhibit excellent responses, and this pH was selected as optimal.

#### 3.1.6. Optimization of the number of gel and IL composite migrations into the tube

The number of times the gel is migrated through the analyte's solution determines the contact time and the extent of IL release. The effect of migration cycles (1–15) was studied (Fig. 3a). The responses increase sharply from 1 to 9 cycles for both analytes due to the more contact area of the IL and sample solution. At 10 cycles, a slight decrease is observed for enrofloxacin, possibly due to the increasing its solubility in the sample solution. Therefore, 9 migration cycles were selected as the optimum.

**Fig. 3:**
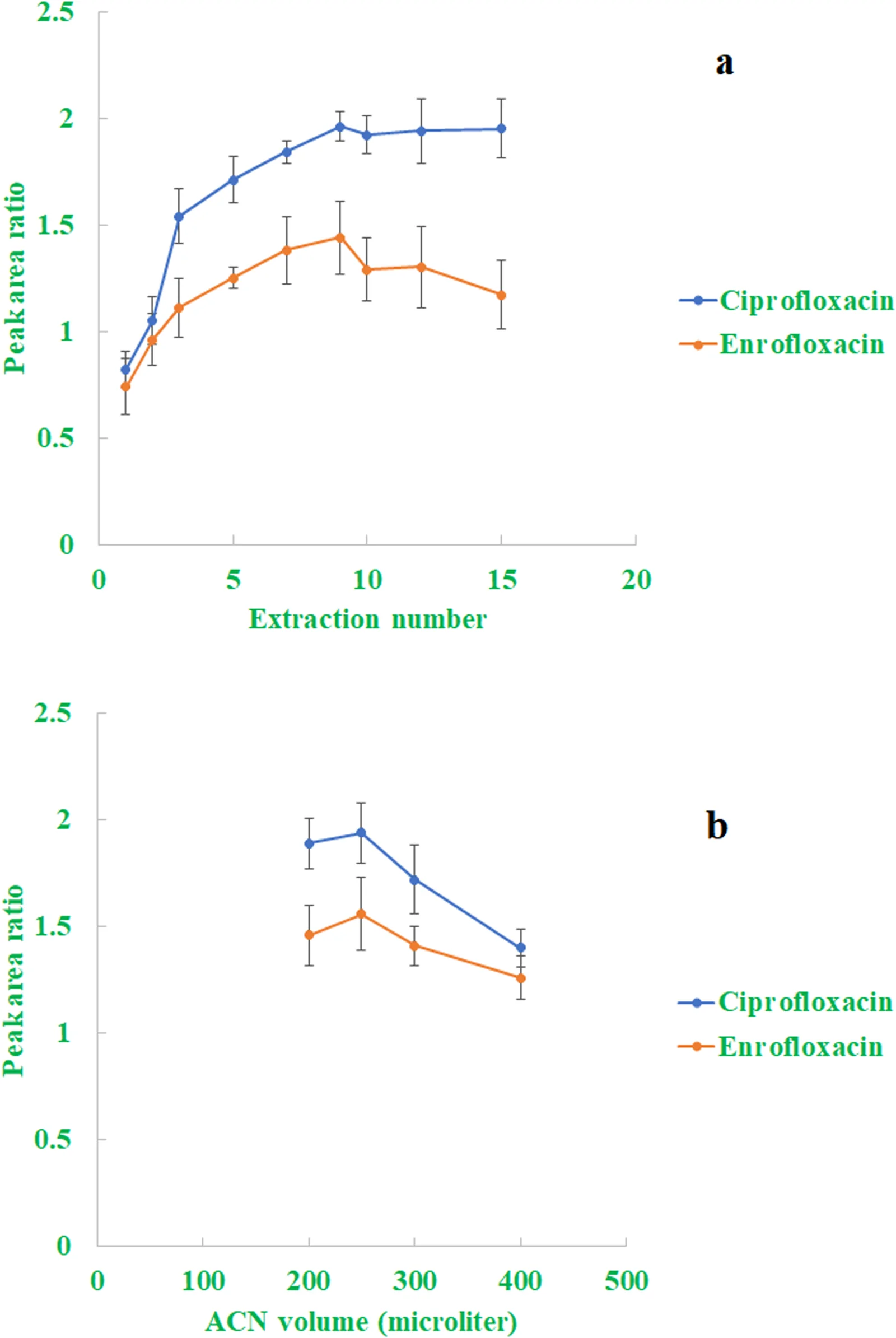
(a) Optimization of the numbers of extraction (gel migration). Extraction conditions: pH 4, and other conditions were described in Fig. 2c. The analytical response was defined as the ratio of the peak area of each analyte to the peak area of the IS. The standard deviation of three replicate analyses was used for error bar calculations. (b) Optimization of acetonitrile volume. Extraction conditions: number of extraction (gel migration): 9, and other conditions were described in (a). The analytical response was defined as the ratio of the peak area of each analyte to the peak area of the IS.

#### 3.1.7. Optimization of acetonitrile (collecting solvent) volume

The volume of acetonitrile as the solvent for collection of the IL containing the analytes should be chosen to ensure complete recovery of the analytes, no excessive dilution and loss of sensitivity. In this study, the effect of acetonitrile volume in the range of 200 to 400 µL on extraction efficiency was investigated (3b). The results show that increasing the acetonitrile volume from 200 to 250 µL increase the responses for both analytes. This increase can be attributed to complete collecting of IL droplets enhancing the method efficacy. With increasing the volume to 300 and 400 µL, the response for both analytes decrease significantly, which is mainly due to increasing the solubility of the analytes and IL reducing of the final analyte concentration and the method efficiency. Therefore, 250 µL of acetonitrile was selected as the optimal volume, which ensures complete elution while avoiding excessive dilution.

## 4. Validation of the proposed method

To thoroughly assess the analytical performance of the proposed method, a series of validation parameters including linear range (LR), coefficient of determination (*r*^2^), limit of detection (LOD), limit of quantification (LOQ), ER, and precision (expressed as relative standard deviation), (RSD%) were determined under optimized experimental conditions (Table 1). The LOD and LOQ values were calculated from signal-to-noise (S/N) ratios of 3 and 10, respectively, where the signal was defined as the peak height and the noise as the standard deviation of the baseline. All peaks were integrated using the instrument software with automatic baseline correction, applying the same integration parameters for all samples and standards. The baseline was fitted over the retention window of each analyte and manually inspected to ensure correct assignment and to avoid baseline drift or mis-integration. The calculated LODs for ciprofloxacin and enrofloxacin were 0.09 and 0.06 ng mL^−1^, while the corresponding LOQs were 0.29 and 0.19 ng mL^−1^, respectively, reflecting the high sensitivity of the developed method. The method exhibited excellent linearity over concentration ranges of 0.30–150 ng mL^−1^ for ciprofloxacin and 0.20–150 ng mL^−1^ for enrofloxacin, with *r*^2^ of 0.991 and 0.985, respectively, indicating a strong linear correlation between the analyte concentration and detector response. The ERs obtained for ciprofloxacin and enrofloxacin were 79 ± 4% and 87 ± 5% (*n* = 3), respectively, demonstrating satisfactory performance of the proposed method in recovering the target compounds. The intra- and inter-day precision of the method was evaluated at three spiking levels (1, 10, and 25 ng mL^−1^ of each analyte) by calculating RSD. The intra-day RSD values (*n* = 6) for ciprofloxacin at these concentration levels were 5.3%, 4.1%, and 3.7%, respectively, and for enrofloxacin were 4.2%, 3.7%, and 3.6%, respectively. The corresponding inter-day RSD values (*n* = 5) for ciprofloxacin were 5.8%, 4.6%, and 3.9%, and for enrofloxacin were 4.4%, 3.9%, and 3.7%, respectively. All obtained RSD values confirm the desirable repeatability and reliability of the method across a broad concentration range. To evaluate the method robustness a series of experiments were conducted at the various values of acetonitrile (240, 250, and 260 µL), pH (3.8, 4.0, and 4.2), salt concentration (1.8, 2.0, and 2.2% w/v), and sample solution temperature (83, 85, and 87 °C). The results obtained showed that the data were consistent with not significant difference observed between them. The variations of the results were ≤16%.

**Table 1:** Quantitative features of the developed method for the determination of ciprofloxacin and enrofloxacin

Analyte	LR^a^	LOD^b^	LOQ^c^	*r* ^2^ d	eRSD%						ER ± SD^f^	
					Intra-day precision at the concentrations (ng mL^−1^) of			Inter-day precision at the concentrations (ng mL^−1^) of				
					1	10	25	1	10	25		
Ciprofloxacin	0.29–150	0.09	0.29	0.991	5.3	4.1	3.7	5.8	4.6	3.9	79 ± 4	
Enrofloxacin	0.19–150	0.06	0.19	0.985	4.2	3.7	3.6	4.4	3.9	3.7	87 ± 5	

a Linear range (ng mL^−1^) (*n* = 7).

b Limit of detection (S/N = 3) (ng mL^−1^).

c Limit of quantification (S/N = 10) (ng mL^−1^).

d Coefficient of determination.

e Relative standard deviation for intra- (*n* = 6) and for inter-day (*n* = 5) precisions.

f Extraction recovery ± standard deviation (*n* = 3).

## 5. Analysis of real samples and evaluation of matrix effect

To evaluate the performance of the proposed method in confronting complex environmental samples and to validate its accuracy under real-world conditions, the optimized protocol was applied to four aqueous samples with distinct origins: pharmaceutical wastewater, urban tap water, river water, and dam water. To evaluate the interference of matrix components on the analytical responses, the samples were spiked with each analyte at two concentration levels (5 and 25 ng mL^−1^) and extracted using the proposed method. In parallel, spiked deionized water samples at the same concentrations were also analyzed. The relative recoveries were calculated as the ratio of the analyte-to-IS peak area ratio in the real samples to that in the deionized water samples at the same concentrations (expressed as a percentage). The results obtained from the analysis of spiked samples are presented in Table 2. As observed, the RR values for the target analytes ranged from 89% to 107%. All these values fall within the acceptable range of 70–120%. Low standard deviations (2–8%) indicate desirable repeatability of the method in different matrices. The highest RR was for ciprofloxacin in tap water at the spiked level of 25 ng mL^−1^ level (107 ± 5%) and the lowest for enrofloxacin in dam water at the spiking level of 5 ng mL^−1^ (89 ± 3%). These observations indicate the absence of significant matrix interference and satisfactory performance of the method in various water samples with different compositions. Additionally, the background concentration of the analytes in the un-spiked samples was also measured. The results showed that ciprofloxacin was only detectable in the pharmaceutical wastewater sample with a concentration of 66 ± 8 ng mL^−1^ (*n* = 3), while enrofloxacin was not detectable in this sample. In other samples (tap water, river water, and dam water), no analytes were found at detectable levels. These findings indicate significant contamination resulting from pharmaceutical factory effluents, which can lead to the introduction of considerable amounts of antibiotics into aquatic environments.

**Table 2:** Matrix effect study

Sample	Mean relative recovery ± standard deviation (*n* = 3)	
	Ciprofloxacin	Enrofloxacin
**All samples were spiked with each analyte at a concentration of 5 ng mL** ^**−1**^		
Wastewater	93 ± 5	98 ± 5
Tap water	107 ± 2	92 ± 7
River water	98 ± 3	93 ± 4
Dam water	92 ± 4	89 ± 3

**All samples were spiked with each analyte at a concentration of 25 ng mL** ^**−1**^		
Wastewater	95 ± 8	96 ± 4
Tap water	92 ± 3	92 ± 2
River water	105 ± 2	96 ± 8
Dam water	107 ± 5	95 ± 2

## 6. Comparison of the studied method with other studies

The analytical performance of the proposed method including of LOD, LOQ, LR, RSD, RR, ER, sample preparation time, and sample size were compared with previously reported techniques for the determination of fluoroquinolone antibiotics in various matrices and the data presented in Table 3.^24–28^ The proposed method demonstrates LODs (0.06–0.09 ng mL^−1^) and LOQs (0.19–0.29 ng mL^−1^) that are fully comparable to those of more sophisticated and costly approaches, while being markedly superior to some other methods. The LR (0.2–150 ng mL^−1^) is sufficiently broad for environmental monitoring purposes, and the precision (RSD < 6%) confirms excellent repeatability. The good and comparable RR% values indicate the independence of the method from matrix interferences. Notably, the proposed method offers several distinct advantages over conventional techniques. Firstly, it eliminates the need for expensive instrumentation such as mass spectrometer or equipment like vacuum manifolds and centrifuge, making it highly accessible for routine laboratories. Secondly, consumption of organic solvent is low (only 250 µL of acetonitrile per each extraction), which is orders of magnitude lower than conventional SPE and LLE methods, thereby significantly reducing the environmental footprint and operational costs. Thirdly, the extraction procedure is operationally simple, rapid, and requires no complex conditioning or multiple extraction steps. The controlled-release mechanism of the IL from the agarose–glycerol matrix upon heating facilitates effective extraction without vigorous agitation or prolonged extraction times. It is notable that short sample preparation duration, higher or comparable ER values, small sample size needing for analysis are the other advantages of the method compared to others.

**Table 3:** Comparison of the proposed method with similar studies

Method	Analyte	Sample	LOD^a^ (ng mL^−1^)	LOQ^b^ (ng mL^−1^)	LR^c^ (ng mL^−1^)	dRSD%	Sample preparation time (min)	ER^e^ (%)	RR^f^ (%)	Sample size	Ref.
DLLME-HPLC-FLD^g^	Enrofloxacin	Surface water	0.11	0.35	—	≤2	>7	70	27–120	8 mL	24
DES-LLME-HPLC-PDA^h^	Ciprofloxacin	Wastewater	0.09	3.0	3–10000	3–11	>20	42	89–109	10 mL	25
	Enrofloxacin		0.09	0.31	0.3–10000	3–7		66	82–122		
MIM-UHPLC-TUV^i^	Ciprofloxacin	Egg	0.7	2.7	5–5000	4.7–6.8	>48	—	89–97	5 g	26
	Enrofloxacin		0.3	1.25	5–5000	1.9–6.0			84–92		
EA-DES-HF-LPME-HPLC-PDA^j^	Ciprofloxacin	Milk	0.3	0.43	0.43–150	≤3.9	>8	—	—	5 mL	27
	Enrofloxacin		0.13	0.78	0.78–150	≤3.4		—	—		
Mixed hemimicelles SPE-HPLC-UV^k^	Ciprofloxacin	Environmental water	0.5	0.05	0.5–200	2.1	>10	—	80–117	10 mL	28
	Enrofloxacin		0.1	0.05	0.1–200	5.5		—	80–120		
AGIL-LPME-HPLC-PDA^l^	Ciprofloxacin	Water and wastewater	0.06	0.29	0.29–150	≤5.3	>3	79	92–107	5 mL	Current work
	Enrofloxacin		0.09	0.19	0.19–150	≤4.2		87	89–98		

a Limit of detection.

b Limit of quantification.

c Linear range.

d Relative standard deviation.

e Extraction recovery.

f Relative recovery.

g Dispersive liquid–liquid microextraction-high performance liquid chromatography-fluorescence detector.

h Deep eutectic solvent–liquid–liquid microextraction-high performance liquid chromatography-diode array detector.

i Molecularly imprinted membrane-ultra high-performance liquid chromatography-tunable ultraviolet detector.

j Effervescent assisted-deep eutectic solvent-hollow fiber liquid-phase microextraction-high performance liquid chromatography-diode array detector.

k Mixed hemimicelles solid phase extraction – high performance liquid chromatography – ultraviolet detection.

l Agarose glycerol ionic liquid–liquid phase microextraction-high performance liquid chromatography – photodiode array.

## 7. Conclusions

In this investigation, a straightforward, rapid, and environmentally sustainable extraction protocol was established by utilizing an agarose–glycerol composite gel as a thermoresponsive carrier for the IL [OMIM][PF_6_]. Upon thermal stimulation, the gel releases the IL, which subsequently acted as the extracting phase for the isolation of ciprofloxacin and enrofloxacin from aqueous matrices. The quantitative validation of the method yielded highly satisfactory performance metrics, including excellent sensitivity with LODs as low as 0.06–0.09 ng mL^−1^, broad LRs extending up to 150 ng mL^−1^, good precision evidenced by RSDs below 6%, and ERs in the range of 79–87%. When applied to real environmental water samples, the method afforded RRs between 89 and 107%, thereby confirming its resilience against matrix interferences. The proposed procedure offered distinct operational benefits, such as experimental simplicity, cost-effectiveness, negligible organic solvent usage, and seamless compatibility with HPLC-PDA instrumentation. Collectively, these attributes positioned the developed approach as a green, practical, and reliable alternative for routine surveillance of fluoroquinolone residues in aquatic environments.

## Conflicts of interest

There are no conflicts to declare.

## Data Availability

The data are available and sharable upon reasonable request.
